# Systematic review and citation content analysis of the CHIME framework for mental health recovery processes: recommendations for developing influential conceptual frameworks

**DOI:** 10.33137/jrmh.v6i1.38556

**Published:** 2023-01-06

**Authors:** Laurie Hare-Duke, Ashleigh Charles, Mike Slade, Stefan Rennick-Egglestone, Ada Dys, Daan Bijdevaate

**Affiliations:** 1 School of Health Sciences, Institute of Mental Health, University of Nottingham, Nottingham, United Kingdom; 2 Nord University, Faculty of Nursing and Health Sciences, Health and Community Participation Devision, Namsos, Norway

**Keywords:** CHIME, mental health recovery, conceptual framework

## Abstract

**Objectives:**

To identify design features of the CHIME conceptual framework of mental health recovery which are associated with high rates of citation.

**Research Design and Methods:**

Systematic review of all citations of the Connectedness, Hope, Identity, Meaning, and Empowerment (CHIME) framework of mental health recovery. Papers citing CHIME were screened and extracted from three citation databases. Citation content analysis was used to investigate associations between nine CHIME design features. Citations were investigated across six forms of visibility: all citations; Anglophone vs non-Anglophone; academic vs non-academic; academic discipline; professional group; and clinical population.

**Results:**

There were 915 eligible documents identified. Six CHIME framework design features met predefined thresholds for high levels of influence: (i) using a systematic review methodology for development, (ii) adopting a memorable acronym, (iii) having disaggregable components, and being unaligned to a (iv) particular discipline (i.e., transdisciplinary), (v) professional group, or (vi) diagnostic population. Documents from Anglophone countries were more likely to cite CHIME with reference to trans-professional (χ2=3.96, df=1, p=0.05) and ethnicity sub-group analysis (p=0.039) design features than non-Anglophone documents. Non-academic documents were more likely to cite the acronym design feature than academic papers (χ2=5.73, df=1, p=0.01). Public Health-related publications were more likely to cite CHIME within a trans-diagnostic framework (χ2=16.39, df=1, p<0.001) than other disciplines.

**Conclusions:**

The influence and impact of conceptual frameworks for recovery are increased when the framework is underpinned by a systematic review, includes disaggregable components which can be summarized using a memorable acronym, and when the framework is transdisciplinary, trans-professional, and trans-diagnostic.

## Introduction

A recovery orientation is now recommended internationally by the World Health Organization.^1^ Despite this policy consensus, the mental health system has been slow to transform. One obstacle is the absence of an operationalised definition of recovery and a shared understanding of the phenomenon.^2,3^ A core difficulty in reaching this shared understanding, however, rests in the inherently idiosyncratic nature of the recovery process.^4^ The challenge is to find a means of integrating the unique elements of recovery into a single foundation that can serve as the platform for the development of mental health interventions, practice, and policy across different contexts.

One approach to support a shared understanding of complex phenomena such as recovery is through the development of conceptual frameworks. A conceptual framework (CF) is “*a network...of interlinked concepts that together provide a comprehensive understanding of a phenomenon*”.^5^

A prominent conceptual framework of recovery is the CHIME framework.^6^ This framework comprises three components: (a) 13 identified characteristics of the recovery journey; (b) five recovery processes comprising: connectedness; hope and optimism about the future; identity; meaning in life; and empowerment (giving the acronym CHIME); and (c) recovery stage descriptions which mapped onto the transtheoretical model of change.^7^

Since its publication, the CHIME framework has been widely cited, with over 2,000 citations (Google Scholar, accessed 26 February 2022). This places it in the top 1% of the Psychology/Psychiatry academic field, according to Clarivate Analytics InCites™ Essential Science indicators. Citation count is the most frequently used approximation of influence,^8^ so CHIME can be regarded as an exemplar influential conceptual framework. The CHIME framework has thus supported a degree of shared understanding around the concept of recovery.

This paper will explore whether there are lessons which can be learnt from the success of the CHIME Framework to help progress other areas of interest within mental health recovery research, and where a lack of a shared understanding might impede their dissemination and adoption within mental health services.^9^ An example is citizenship, a term loosely referring to a range of constructs, from a person having a legal status as a citizen of a country to the person perceiving themselves as a member of society.^10^ There are also concepts embedded within CHIME itself, such as Connectedness, which merit further elaboration to support the implementation of recovery into mental health services.^11^

The CHIME framework had nine design features (DFs) intended to increase impact. This paper will evaluate whether these DFs are associated with the high level of influence of CHIME. These nine DFs are shown in Box 1.

It is not clear whether the CHIME design features have contributed to the influence of this conceptual framework. Particular design features were employed to increase the influence of CHIME within a certain domain. For example, the acronym was used to increase the accessibility of CHIME in non-academic publications. The systematic review methodology was intended to increase the scientific credibility of the framework within academic literature, whether in peer-reviewed journals or a higher education teaching context. The ethnicity sub-group analysis and international validation study were intended to increase the cross-cultural applicability of the framework outside of Anglophone (English-speaking) countries. However, it is not clear which if any of these various strategies were successful.

## Aims and objectives

The aim of this study is to inform the development of recovery-related conceptual frameworks which have maximum influence, using citation as a proxy metric for influence. This aim will be addressed by conducting a citation content analysis of the CHIME framework. The objectives of the citation content analysis are to investigate the relationship between the CHIME design features and the purpose of citation in relation to six different forms of visibility:

Objective 1: Across all citations.

Objective 2: In Anglophone versus non-Anglophone countries.

Objective 3: In academic versus non-academic documents, indicating the framework is accessible beyond academic publications.

Objective 4: In different academic disciplines, indicating the framework transcends disciplinary boundaries.

Objective 5: In publications about different professional groups, indicating the framework is trans-professional.

Objective 6: In studies of different clinical populations, indicating the framework is trans-diagnostic.

These objectives were chosen to investigate key questions relating to recovery as conceptualized within CHIME: Does recovery have cross-cultural validity (Objective 2)? Does recovery make sense to mental health stakeholders beyond the research community (Objective 3)? Is recovery seen as specific to an academic discipline (Objective 4), a professional group. (Objective 5) or a particular diagnosis (Objective 6)?

## Research Design and Methods

### Design

A citation content analysis of all published documents was conducted citing the CHIME framework.^6^ Citing documents were analyzed using a modified version of Citation Content Analysis (CCA).^16^ Modified CCA uses qualitative syntactic analysis (how text is presented and ordered, including word frequencies and the order of elements) and semantic analysis (the meaning of the data). This helps characterize both how often and why a study is cited.

### Search strategy

The following citation databases were searched: Scopus, Web of Science and Google Scholar. These databases span a range of scientific databases,^17^ have been previously used in CCA studies,^18^ and combining multiple databases is recommended for citation tracking.^18^ Whilst the CHIME framework was originally published in a psychiatric journal, the search strategy was intended to track the influence of CHIME both within and outside of health and recovery research, and usage of these database was appropriate due to their broad disciplinary coverage of research outputs. Inclusion criteria were: (1) citation of the CHIME framework; (2) Document is available in English; and (3) full text of the document is available. Exclusion criteria were: (1) citation of the CHIME framework in the reference list but not in the main text; and (2) citation record is not a document. The search was conducted in each database from inception until 25 November 2019.

### Procedure

All references which included a citation of the CHIME framework were stored in EndNote version 9, which was selected due to its availability and familiarity to all members of the review team. Duplicates were removed, initially automatically and then manually. Full text documents of remaining references were retrieved, and screened against inclusion and exclusion criteria by AC, AD, DB and LHD. Throughout, interim versions of EndNote files were regularly archived to a shared research file system in case of database corruption.

A codebook was developed to specify numeric (e.g. how often cited), literal (e.g. position in the citing manuscript) and sociocultural data (e.g. reason why the citation is made) to be abstracted. Descriptive information was also collected for the citing document. The codebook was developed through iterative piloting. The preliminary codebook comprised selected categories suggested in the CCA methodology paper (type of cited document, type of authorship, relation to the citing work, location of mentioning, function of citation, disposition of citation),^16^ augmented with categories commonly used in systematic review data abstraction tables to describe included documents, such as country of first author and type of study. The preliminary codebook was iteratively refined through piloting with 20 randomly selected included documents by AD and DB, followed by piloting with a different 60 randomly selected included documents by AD and DB, with disagreement resolved through discussion and refinement of the coding procedure. The final codebook achieved 100% concordance. Finally, new categories were included following discussion within the review team (AC, LHD, MS, SRE). The final codebook identifies the information extracted from each included document about the CHIME Framework citation, and is shown in Table 1.

Once the codebook was finalized, data from all included papers were extracted by AC, AD, DB, and LHD to the Data Abstraction Table which contained headings as per the codebook.

### Analysis

The CHIME design features were operationalized as follows. DF1.1 (Acronym): reference to the ‘CHIME’ acronym. DF1.2 (Disaggregable components): reference to one or more of the individual CHIME components (Connectedness, Hope, Identity, Meaning, Empowerment). DF2.1 (Systematic review): reference to the CHIME systematic review development methodology. DF3.1 (Transdisciplinary): documents published in journals explicitly targeting a transdisciplinary audience or non-journal documents which describe a transdisciplinary focus. DF3.2 (Trans-professional): documents published in journals targeting a trans-professional audience or non-journal documents which describe a trans-professional focus. DF3.3 (Trans-diagnostic): documents published in journals with an explicit trans-diagnostic approach or non-journal documents which use a trans-diagnostic approach.

To meet Objective 1 (all citations), univariate descriptive statistics were calculated for each design feature across all citations. Establishing thresholds is a key procedure to examine the influence of documents using CCA.^19^ For the purpose of analyzing the rate of citation of each design feature, a frequency of less than 1 in 10 citations was arbitrarily taken to indicate a low rate, under 1 in 4 indicating a medium rate, and 1 in 4 or above representing a high rate. Therefore, thresholds for low, medium, and high rates of citation were set at 0-9%, 10-24%, and 25% or more respectively.

To meet Objective 2 (Anglophone versus non-Anglophone citations), the design features were cross-tabulated with Anglophone and non-Anglophone documents. This was operationalized as a binary variable dichotomising the country of affiliation of the lead author of a document into English-speaking (Anglophone) and non-English speaking (non-Anglophone, e.g. countries where English is not an official language). Bivariate associations between each type of document and the CHIME design features were analyzed using chi-square tests (α<0.05). Variables with more than two possible values (e.g., the design feature DISAGGREGABLE COMPONENTS has Connectedness, Hope, Identity, etc.) were analyzed with post-hoc tests where the initial bivariate test was significant. For these analyses, post-hoc tests were conducted using adjusted residual analysis to compare the observed frequencies of citations with the expected frequencies for each cell. To correct for multiple testing, the post-hoc analyses used the Bonferroni correction, which adjusts the significance value by the number of tests conducted. Fisher’s Exact Test (FET) was used for comparisons with small (<5) expected cell counts.

To meet Objectives 3 to 6, the same analysis as was used for Objective 2 was conducted for Objective 3 (comparing Academic versus non-Academic citations instead of Anglophone versus non-Anglophone citations), Objective 4 (comparing citations across disciplines), Objective 5 (citations across professional groups), and Objective 6 (citations across clinical populations).

Citations of the design features were also compared across sole citations (i.e., CHIME cited alone) versus joint citations (CHIME cited in a group with other citations, such as other publications presenting knowledge about mental health recovery processes) as it was expected that influential design features were more likely to be referenced as sole citations. All analyses were conducted using Stata 16.

## Results

**Figure 1:**
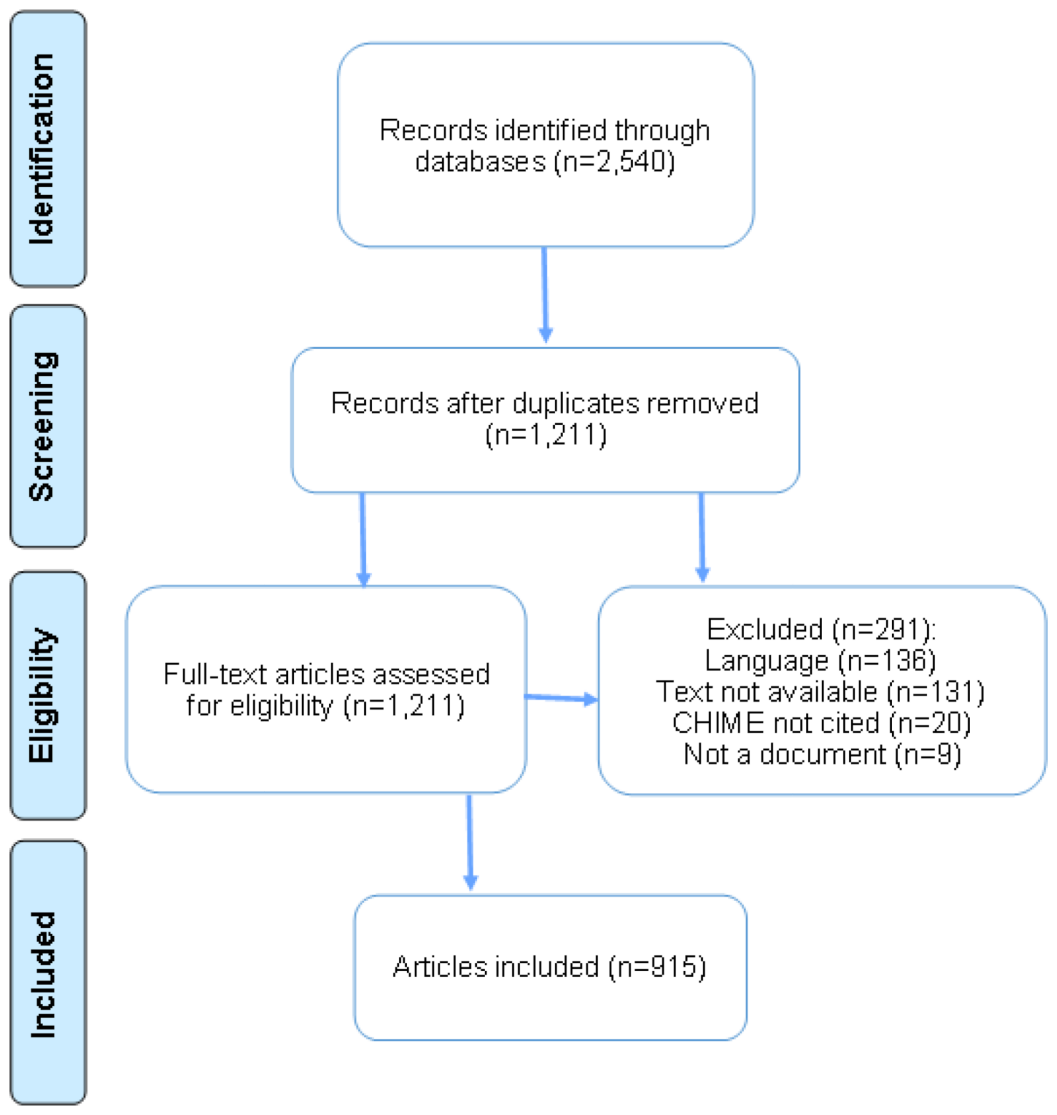
Flow diagram for included publications

In total, 1,211 unique documents were identified, of which 915 were included. The flow diagram for included documents is shown in Figure 1.

The overall sample included 896 (98%) academic papers. Country of citation was varied (UK n=222; Australia n=121; Canada n=48; USA n=46; Norway n=41; Netherlands n=29) with 469 (49%) of papers from non-Anglophone countries. The rate of citation increased each year, from 56 citations in 2012 to 197 in 2018. The purpose of citation was as follows: describing what comprises recovery (n=917, 51%), highlighting a study limitation (n=275, 15%), justifying research topic (n=139, 8%), highlighting a research gap (n=105, 6%), providing a practice recommendation (n=47, 3%), informing the study methodology (n=39, 2%), supporting research findings (n=16, 1%), providing a recommendation for future research (n=10, 1%), contradicting study findings (n=4, <1%), providing a definition of recovery (n=24, 1%), and Other (n=213, 12%). Overall, 96% of citations made a correct reference to the CHIME framework, i.e., did not cite the framework in an incorrect or biased manner. Supplementary File 1 contains the Data Abstraction Table, giving a full description of all included documents using the categories shown in Table 1.

### Objective 1 (All citations)

The frequency of citations relating to each CHIME framework design feature is shown in Table 2.

There were six highly cited design features: DF1.1 (Acronym), DF1.2 (Disaggregable components), DF2.1 (Systematic review), DF3.1 (Transdisciplinary), DF3.2 (Trans-professional) and DF3.3 (Trans-diagnostic). This indicates that the high citation of the CHIME Framework is primarily based on the acronym, credibility as a systematic review, and neutrality in relation to academic discipline, diagnosis, and profession.

Sole citations were more likely to cite DF1.1 (Acronym) (χ^2^=84.15, df=1, p<0.001), DF1.2 (Disaggregable components) (χ^2^=88.36, df=6, p<0.001) and DF2.1 (Systematic review) (χ^2^=49.00, df=1, p<0.001) than joint citations. There were no other differences between sole and joint citations.

In terms of the purpose of citation, papers which referred to the DF2.1 (Systematic review) (χ2=13.25, df=1, p<0.001) and *all components* of DF1.2 (Disaggregable components) (χ2=58.92, df=1, p<0.001) were more likely to cite the CHIME framework in order to describe what comprises recovery. Papers which cited the *Connectedness* (χ2=32.86, df=1, p<0.001) or *Identity* (χ2=9.26, df=1, p<0.001) components of DF1.2 (Disaggregable components) design feature were less likely to cite CHIME in order to highlight a limitation of the study.

### Objective 2 (Anglophone versus non-Anglophone)

Authors of papers from Anglophone countries were more likely to cite CHIME with reference to the DF3.2 (Trans-professional) (χ2=3.96, df=1, p=0.05) and DF2.2 (Ethnicity) (p=0.039; FET) design features than authors from non-Anglophone countries. There were no significant differences between these types of publications in relation to any of the other design features. If the CHIME Framework is viewed as a proxy for recovery, this indicates that the cross-cultural validity of recovery is increased by the ethnicity sub-group analysis.

### Objective 3 (Academic versus non-academic)

Non-academic papers were more likely to cite the DF1.1 (Acronym) design feature than academic papers (χ2=5.73, df=1, p=0.01). There were no significant differences between academic and non-academic publications in relation to any of the other design features. If the CHIME Framework is viewed as a proxy for recovery, this indicates that the use of an acronym increases the accessibility of recovery to non-academic audiences.

### Objective 4 (Transdisciplinary)

Public Health publications were more likely to cite CHIME within a transdiagnostic framework (χ2=16.39, df=1, p<0.001) than other disciplines. There were no other significant differences between academic disciplines in the citation of design features. If the CHIME Framework is viewed as a proxy for recovery, this indicates that recovery is not viewed as specific to one academic discipline.

### Objective 5 (Trans-professional)

There were no significant differences between professional groups in relation to any design feature. If the CHIME Framework is viewed as a proxy for recovery, this indicates that recovery is not viewed as specific to one professional group.

### Objective 6 (Trans-diagnostic)

There were no significant differences in the citation of design features between papers focussing on different diagnostic groups. If the CHIME Framework is viewed as a proxy for recovery, this indicates that recovery is not viewed as specific to one diagnostic group.

## Conclusions

This review identified six highly-cited design features of the CHIME framework which may inform the development of other recovery-related frameworks to have maximum impact. These comprise: (i) using a systematic review methodology, (ii) adopting a memorable acronym, (iii) having disaggregable components, and being unaligned to a particular (iv) discipline, (v) professional group, or (vi) diagnostic population.

The systematic review design feature was associated with citations used to describe what comprises recovery. Like many psychosocial constructs, the meaning of mental health recovery is complex and contested.^20^ The findings from this study suggest that conceptual frameworks attempting to describe such constructs may have a greater influence if they are developed using robust methodologies, such as systematic reviews.

The CHIME framework uses an easily accessible acronym instead of being drawn from terminology used within health services research. In this study it was found that non-academic papers were more likely to cite the acronym design feature than academic papers. Recovery-related frameworks are intended to have an impact outside of an academic context, particularly in a clinical context, but also in areas such as legal settings or in public policy.^21,22^ It may be recommended that conceptual frameworks use accessible language, rather than technical jargon, in order to maximize their impact outside of academia. Developers of conceptual frameworks may therefore need to consider how to select an acronym without distorting the conceptual content of frameworks.

The CHIME framework was developed by UK-based researchers and drew on data collected from predominantly Anglophone countries. However, the results of this study suggest that CHIME has had cross-cultural influence, with approximately half of the citing papers being written by authors from non-Anglophone countries. The components of the CHIME framework – Connectedness, Hope, Identity, Meaning, and Empowerment – have each been described as universal psychosocial needs.^23–26^ In this study there were no differences between Anglophone and non-Anglophone papers in the frequency with which each of these components were cited. One potential implication of these findings is that conceptual frameworks may have most impact when they use abstract, cross-cultural concepts which can be readily adapted for local contexts.

Three design features were not found to be related to the high impact of the CHIME framework and cannot be recommended for the purpose of increasing the visibility of conceptual frameworks within psychiatric rehabilitation: independent and non-independent validation studies and the ethnic minority sub-group analysis. All of the CHIME validation studies used qualitative methods. Different findings may have emerged for quantitative validation studies which are seen as being more robust within certain disciplines. Future studies of influential conceptual frameworks could examine this distinction between qualitative and quantitative validation studies. Whilst it was found that the ethnic minority sub-group analysis was not related to the overall level of influence of CHIME, such methods may be used for other scientific and ethical reasons in establishing the applicability of conceptual frameworks across different groups.

This is the first study to use CCA to assess the influence of a conceptual framework and thereby establishes the possibilities of this method. A previous CCA study of an influential paper concluded that the impact of the paper was largely due to citation bias.^27^ Incorrect interpretations and reporting are known to be an issue for citations to specific documents^28^ as well as for general knowledge claims based upon a set of key influential papers.^29,30^ By contrast, CHIME was correctly cited by the overwhelming majority of papers, which allowed for analysis of its influential design features. As it is unclear to what extent other conceptual frameworks are cited correctly, one methodological recommendation is that the accuracy of citations should be assessed as part of any future CCA studies.

The six design features recommended for the development of conceptual frameworks were each cited in approximately 30% or more of the citations collated in this review where CHIME was the sole citation (i.e., not with other citations). In addition, there was a significant difference between sole and joint citations in relation to three of these design features. A second methodological recommendation of this study is therefore that future CCA studies should similarly compare sole and joint citations where relevant.

### Strengths

This is the first paper to use CCA to assess the influence of a conceptual framework. It has been shown to be possible to analyze the influence of a conceptual framework in terms of its design features, and future CCA studies may employ a similar methodology.

This study employed robust procedures in searching, screening, and extracting the data. First, whilst many CCA studies have been conducted by searching a single database, this study used a more comprehensive approach by searching multiple databases as is now recommended when assessing citations across disciplines.^18^

Second, the screening process for included studies was recorded and reported. Third, the Data Abstraction Table was developed iteratively by multiple authors, piloted, and checked for concordance.

The very high citation rate of the CHIME framework provided a sufficiently large sample to allow detailed analyses of the design features which may have contributed to the influence of this framework.

### Limitations

Citation was used as a proxy metric for influence, but citation in other publications is only one aspect of impact. For example, the UK Research Excellence Framework 2021 define impact as ‘an effect on, change or benefit to the economy, society, culture, public policy or services, health, the environment or quality of life, beyond academia.’ These wider aspects of influence and impact of CHIME were not explored.

CCA was found to be a suitable method of assessing the influence of a conceptual framework in terms of its design features. However, CCA is not a suitable means of analyzing all of the factors which may be relevant to the influence of a conceptual framework, such as the policy context and the national/international profile of the framework authors,^31^ or factors such as cross-citation within the same issue.^32^ For instance, the CHIME framework of mental health recovery was published in the same year that the UK Department of Health published a key policy document emphasizing the importance of recovery,^33^ with 36% of lead authors citing CHIME coming from the UK. This limits the recommendations that can be made on the basis of this study as to the most important factors to consider when developing conceptual frameworks.

One factor which may be important for the influence of conceptual frameworks is the dissemination strategies used by the authors, such as promoting the paper to colleagues or at conferences. The extent to which these factors might have increased the influence of CHIME is unclear from the methods used in this study and further methodological innovation is required to explore this. For example, a network analysis of the authors/co-authors^34^ which cite a conceptual framework might be used to assess the extent to which (i) high citation counts are attributable to clusters of individuals, indicating the extent to which dissemination via other research groups is effective, and (ii) how influence spreads from a lead author on an initial paper to other papers written by their co-authors.

### Implications

This study identifies the importance of design aspects when developing recovery-related knowledge products intended for widespread use. This approach is already visible in the development of pro-recovery interventions. One example of an approach which has become widely used is Individual Placement and Support (IPS), for which a systematic review identified 27 randomized controlled trials.^35^ IPS is now being integrated with other complex interventions, such as Cognitive Remediation Therapy^36^. Similarly, the proposed research agenda for mental health peer support work^37^ has been responded to, with a systematic review identifying 19 randomized controlled trials,^38^ with others ongoing, such as the Understanding Peer Support in Developing Empowering mental health Services (UPSIDES) randomized controlled trial in low and middle income countries.^39^ These intervention examples use acronyms, are underpinned by systematic reviews, and are not aligned with any specific academic discipline, diagnostic, or professional group. In addition to interventions, our study of the CHIME Framework for recovery indicates that this design approach can also be applied to important recovery-related concepts such as hope, empowerment and shared decision-making. For example, in our research group we are using these approaches to develop mental health-related conceptual frameworks for recovery narratives,^40^ spirituality,^41^ and social connectedness.^42^

Non-alignment of the CHIME Framework emerged as particularly important. Perhaps an example of a theoretical framework which has not got wide traction is the Model of Human Occupations,^43^ which is established with and ‘owned’ by occupational therapists, but not greatly used beyond that professional group as a framework for understanding occupation. Future recovery-related frameworks should de-emphasise alignment with particular disciplines, professions, and diagnoses. Coproduction involving people with lived experience of mental health issues is a good approach to achieving this neutrality, which our research group is exploring in relation to citizen science approaches (https://www.researchintorecovery.com/research/c-stacs).

In conclusion, the findings of this study may inform the future development of conceptual frameworks within recovery with a view to maximizing their visibility and influence. Whilst recognising the limitations of citation metrics,^44^ it is recommended that conceptual frameworks are developed using six design features: (i) systematic review methodology, (ii) memorable acronym, (iii) disaggregable components, and being unaligned to a particular (iv) discipline, (v) professional group, or (vi) diagnostic population. These features may be especially important for conceptual frameworks of complex, contested phenomena and for having influence across both academic and non-academic contexts.

## Supplementary Material

Supplementary File 1

## Figures and Tables

**Table 1:** Codebook for data extraction from included documents

Category	Definition [permissible values]

**CHARACTERISTICS OF THE INCLUDED DOCUMENT**	
Reference	Document reference number [Reference]
Country	Country of first affiliation of lead author [Country]
Self-citation	Does the document author list include at least one CHIME framework author [Yes, No]
Region	Scope of data collection (data-based papers) or recommendations (non-data-based papers) [sub-national, national, international, not specified]
Year	Year of publication of online version [Year]
Type	Document type [Journal paper, Conference proceedings, PhD Thesis, Opinion piece/commentary, Policy paper, Non-governmental organization report, Book, Website, Other]
Design	Study design [Randomised controlled trial, Study protocol, Non-systematic review, Systematic review, Other data-based design, Editorial]
Academic	Published in a peer-reviewed journal or student thesis/dissertation [Yes, No]
Intervention	Paper reports development or evaluation of an intervention [Yes, No]
Population	Study sample (data-based papers) or population discussed (non-data-based papers) [Diagnostic, professional, different demographic groups e.g., parents/carers]

**CHARACTERISTICS OF THE CITATION**	
Citation count	Total number of times CHIME is cited in the document [Numeric value]
Citation location	Location of each citation [For data-based papers: Introduction/Background, Methods, Results, Discussion; Conclusions; Strengths and limitations; For non-data-based papers: General introduction/background; General discussion]
Citation location count	Number of times CHIME is cited in the location [Numeric values]
Direct quotation	Verbatim text from one sentence before to one sentence after citation [In-text quotations]
Citation purpose	The purpose of the citation [Describe what recovery means, Justify research topic, Highlight knowledge gap, Inform methodology, Support research findings, Contradict research findings, Highlight a limitation, Provide a practice recommendation for practice, Provide a research recommendation, Provide a definition of recovery, Other]
Sole/joint citation	CHIME citation is on its own or with other citations [Sole, Joint]

**CITATION CONTENT ANALYSIS**	
DF 1.1 (Acronym)	The CHIME acronym was cited [Yes, No]
DF 1.2 (Disaggregable components)	CHIME framework component cited [Connectedness, Hope, Identity, Meaning, Empowerment, Complete CF; None specified]
DF 2.1 (Systematic review)	Reference made to CHIME being developed using systematic review methodology [Yes, No]
DF 2.2 (Ethnicity)	Reference made to the CHIME ethnicity sub-group analysis [Yes, No]
DF 2.3 (Validation studies)	Reference to any validation study (Slade et al., 2012; Bird et al., 2014; Brijnath, 2015, Stuart et al., 2017) [Yes, No]
DF 2.4 (Independent validation studies)	Reference to an independent CHIME validation study (Brijnath, 2015; Stuart et al., 2017) [Yes, No]

**CHARACTERISTICS OF THE JOURNAL AND PUBLICATION**	
DF 3.1 (Transdisciplinary)	Journal is transdisciplinary [Yes, No]
DF 3.2 (Trans-professional)	Journal is trans-professional [Trans-professional, Specific profession, No profession]
DF 3.3 (Trans-diagnostic)	Publication is trans-diagnostic [Trans-diagnostic, Single diagnosis, No population]

**Table 2:** Citation of each CHIME framework design feature (DF) in included documents (n=915)

Design feature referenced when citing CHIME framework n (%)		All citations	Single citations	With citations other
	n	915	718	197
**Acronym**				
Reference to CHIME acronym (DF1.1)				
	*Yes*	**250 (27)**	**247 (34)**	3(2)
	*No*	665 (73)	471 (66)	194 (98)
Reference to disaggregable components (DF1.2)				
		142 (7)	101 (7)	41 (7)
	*Connectedness*	159 (8)	94 (7)	65 (12)
	*Hope*	107 (5)	71 (5)	36 (6)
	*Identity*	81 (4)	59 (4)	22 (4)
	*Meaning*	144 (7)	99 (7)	45 (8)
	*Empowerment*	**605 (31)**	**550 (38)**	55 (10)
	*Complete CHIME*	763 (38)	462 (32)	301 (53)
	*No reference*			
**Scientific quality**				
Reference to systematic review (DF2.1)				
	*Yes*	206 (22)	**198 (28)**	8 (4)
	*No*	709 (77)	520 (72)	189 (96)
Reference to ethnicity sub-group analysis (DF2.2)				
		9 (1)	9 (1%)	0 (0)
	*Yes*	906 (99)	709 (99)	198 (100)
	*No*			
Reference to any validation study (DF2.3)				
	*Yes*	54 (6)	42 (6)	12 (6)
	*No*	861 (94)	676 (94)	185 (94)
Reference to independent validation study (DF2.4) (672 documents published post-2015)				
	*Yes*	11 (2)	10 (2)	1 (1)
	*No*	661 (98)	508 (98)	153 (99)
**Unaligned**				
Citing document is transdisciplinary (DF3.1)				
	*Yes*	**258 (63)**	**209 (63)**	**49 (62)**
	*No*	155 (38)	155 (38)	30 (38)
Citing document is trans-diagnostic (DF3.2)				
	*Yes*	**278 (30)**	**227 (32)**	**51 (26)**
	*No*	574 (63)	444 (62)	130 (66)
	*Not specified*	63 (7)	47 (7)	16 (8)
Citing document is trans-professional (DF3.3)				
	*Yes*	**258 (34)**	**209 (35)**	**49 (29)**
	*No*	502 (66)	384 (65)	118 (71)

Bold = design features with a high (25% or more) rate of citation.

## Box 1 Design Features (DFs) used in the CHIME Framework

### MEMORABLE ACRONYM

DF1 CHIME is easy to spell and remember, with positive connotations and metaphorical associations connected with the CF topic (e.g. “your ideas chime with me” means I connect with your ideas).

DF2 CHIME contains specific disaggregable components (i.e. Connectedness, Hope etc.).

### SCIENTIFIC QUALITY

DF3 CHIME was developed through a methodologically rigourous systematic review.

DF4 The CHIME framework includes a novel ethnicity sub-group analysis.

DF5 The validity of the CHIME framework was subsequently established by the same research team with current mental health service users^12^ and in an international validation study investigating cross-cultural use.^13^

DF6 Further validation studies were conducted by independent research groups.^14,15^

### UNALIGNED

DF7 The CHIME framework is transdisciplinary, not, for example, a psychological or sociological framework of recovery.

DF8 The CHIME framework is trans-professional, not, for example, a psychiatric or nursing framework of recovery.

DF9 The CHIME framework is trans-diagnostic, not, for example, a psychosis or severe mental illness framework of recovery.
